# Effect and mechanism of NaHS on tobacco bacterial wilt caused by *Ralstonia solanacearum*

**DOI:** 10.1038/s41598-022-26697-8

**Published:** 2023-02-11

**Authors:** Dingxin Wen, Qingqing Guo, Wan Zhao, Yong Yang, Chunlei Yang, Jun Yu, Yun Hu

**Affiliations:** 1 State Key Laboratory of Biocatalysis and Enzyme Engineering, College of Life Science, Hubei University, Wuhan, 430062 China; 2 Tobacco Research Institute of Hubei Province, Wuhan, 430030 Hubei China

**Keywords:** Microbiology, Plant sciences

## Abstract

Since its discovery as a third unique gaseous signal molecule, hydrogen sulfide (H_2_S) has been extensively employed to resist stress and control pathogens. Nevertheless, whether H_2_S can prevent tobacco bacterial wilt is unknown yet. We evaluated the impacts of the H_2_S donor sodium hydrosulfide (NaHS) on the antibacterial activity, morphology, biofilm, and transcriptome of *R. solanacearum* to understand the effect and mechanism of NaHS on tobacco bacterial wilt. In vitro, NaHS significantly inhibited the growth of *Ralstonia solanacearum* and obviously altered its cell morphology. Additionally, NaHS significantly inhibited the biofilm formation and swarming motility of *R. solanacearum*, and reduced the population of *R. solanacearum* invading tobacco roots. In field experiments, the application of NaHS dramatically decreased the disease incidence and index of tobacco bacterial wilt, with a control efficiency of up to 89.49%. The application of NaHS also influenced the diversity and structure of the soil microbial community. Furthermore, NaHS markedly increased the relative abundances of beneficial microorganisms, which helps prevent tobacco bacterial wilt. These findings highlight NaHS's potential and efficacy as a powerful antibacterial agent for preventing tobacco bacterial wilt caused by *R. solanacearum*.

## Introduction

Bacterial wilt is a soil-borne disease caused by *Ralstonia solanacearum* that has a global impact on crop quality and productivity^1–3^. Bacterial wilt causes soil degradation, poor plant development, an overabundance of harmful bacteria, and breakouts of soil-borne diseases^4,5^. As a result, it has been attributed to a disturbed ecological balance between plants and rhizosphere microorganisms^6^.

Agrochemicals are frequently used to control bacterial wilt^1,7^. Nevertheless, excessive agrochemical use can have major adverse effects, including the spread of resistant strains and environmental threats^8,9^. Prevention research frequently uses agricultural controls such as resistant cultivars, crop rotation, and tillage control^3,6^. It is challenging to manage *R. solanacearum* through agricultural practices due to its protracted presence in the soil outside the host plant. Hence, there is a pressing need to create new alternative agent compounds that effectively control *R. solanacearum* and are environmentally friendly.

Hydrogen sulfide (H_2_S) is the third unique gaseous signal molecule used in animal physiological processes and agricultural production^4,10^. A large number of studies in the field of plants have shown that H_2_S can be directly or indirectly involved in a wide range of plant physiological processes, including stomatal movement^11^, photosynthesis^12^, seed germination^13^, root growth^14^, fruit ripening^15^, as well as plant senescence^16^. H_2_S can enhance plant tolerance to drought, salinity, high-temperature, and heavy metal stress by initiating plant redox signal, antioxidant capacity, and specific components of cellular defense^17–20^. Exogenous application of H_2_S induces plant cross-adaptation to multiple abiotic stresses^21^. Therefore, H_2_S, a sulfur-containing defense compound, plays an important role in plant resistance to biotic and abiotic stresses^22^. Previous researches have demonstrated that H_2_S prevents the growth of several pathogens, such as *Rhizopus oryzae*, *Aspergillus niger*, *Penicillium italicum*, *Candida albicans*, and *Aspergillus niger*^23,24^. However, it is unknown whether H_2_S can prevent *R. solanacearum* and whether it can decrease tobacco bacterial wilt (TBW) caused by the pathogen *R. solanacearum*.

The compound sodium hydrosulfide (NaHS) is widely used as H_2_S donor in basic research. Upon hydrolysis, NaHS were dissociated to Na^+^ and HS^−^, and then partially binding to H^+^ to form H_2_S^25^. In this study, we evaluated the impacts of the H_2_S donor sodium hydrosulfide (NaHS) on the antibacterial activity, morphology, biofilm, and transcriptome of *R. solanacearum*. In addition, NaHS was administered to a tobacco field infested with bacterial wilt. The effect of NaHS on disease incidence, disease index, rhizosphere soil physicochemical properties, and microbial communities was studied to determine the mechanism by which NaHS works to prevent TBW. These investigations will establish NaHS as a unique and eco-friendly antibacterial agent for the control of TBW.

## Results

### In vitro antibacterial activity of NaHS against *R. solanacearum*

**Figure 1:**
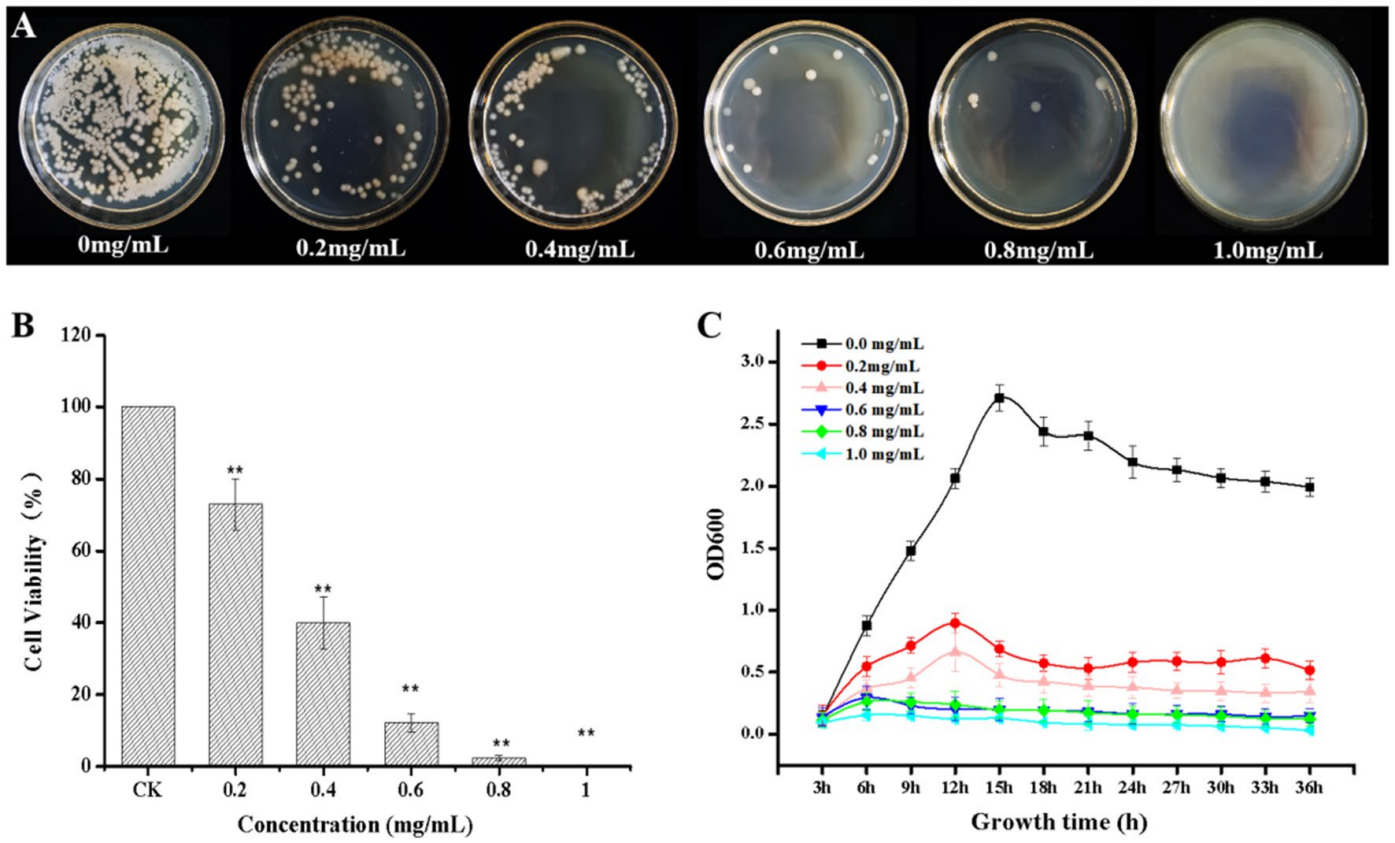
NaHS has an antibacterial effect against *R. solanacearum*. (**A**) The growth of *R. solanacearum* on plates after 24 h of culture. (**B**) The cell viability rate of *R. solanacearum*. (**C**) The OD growth curves of *R. solanacearum* in the presence of different concentrations of NaHS. The error bars represent the standard deviations of three replicates, and a **p-value of < 0.01 indicates statistically significant differences based on independent-sample *t-tests*.

Using NA plates, different concentrations of NaHS were evaluated at 24 h to examine if they may inhibit the development of *R.* *solanacearum* (Fig. 1A). Figure 1A demonstrates how NaHS suppressed *R. solanacearum* growth on plates after 24 h. Additionally, the plate counting method assessed the viability of *R. solanacearum* (Fig. 1B). Cell viability was 73.03%, 40.01%, 12.16%, and 2.30% at amounts of 0.2, 0.4, 0.6, and 0.8 mg/mL, respectively. The cells displayed total inactivation and no viability following treatment with NaHS at a 1.0 mg/mL dosage. This information suggested that NaHS had an antibacterial action that varied with concentration. The growth curves also calculated NaHS's antibacterial efficacy (Fig. 1C). At concentrations of 0.2, 0.4, 0.6, 0.8, and 1.0 mg/mL, NaHS strongly reduced the growth of *R. solanacearum*, and after 12 h of incubation, the *R. solanacearum* growth stopped. Moreover, at concentrations of 0.6, 0.8, and 1.0 mg/mL, *R. solanacearum* growth was almost entirely suppressed. However, NaHS had no effect on the colony shape of *R. solanacearum* (Supplementary Fig. S1).

### The influence of NaHS on the morphology of *R. solanacearum*

**Figure 2:**
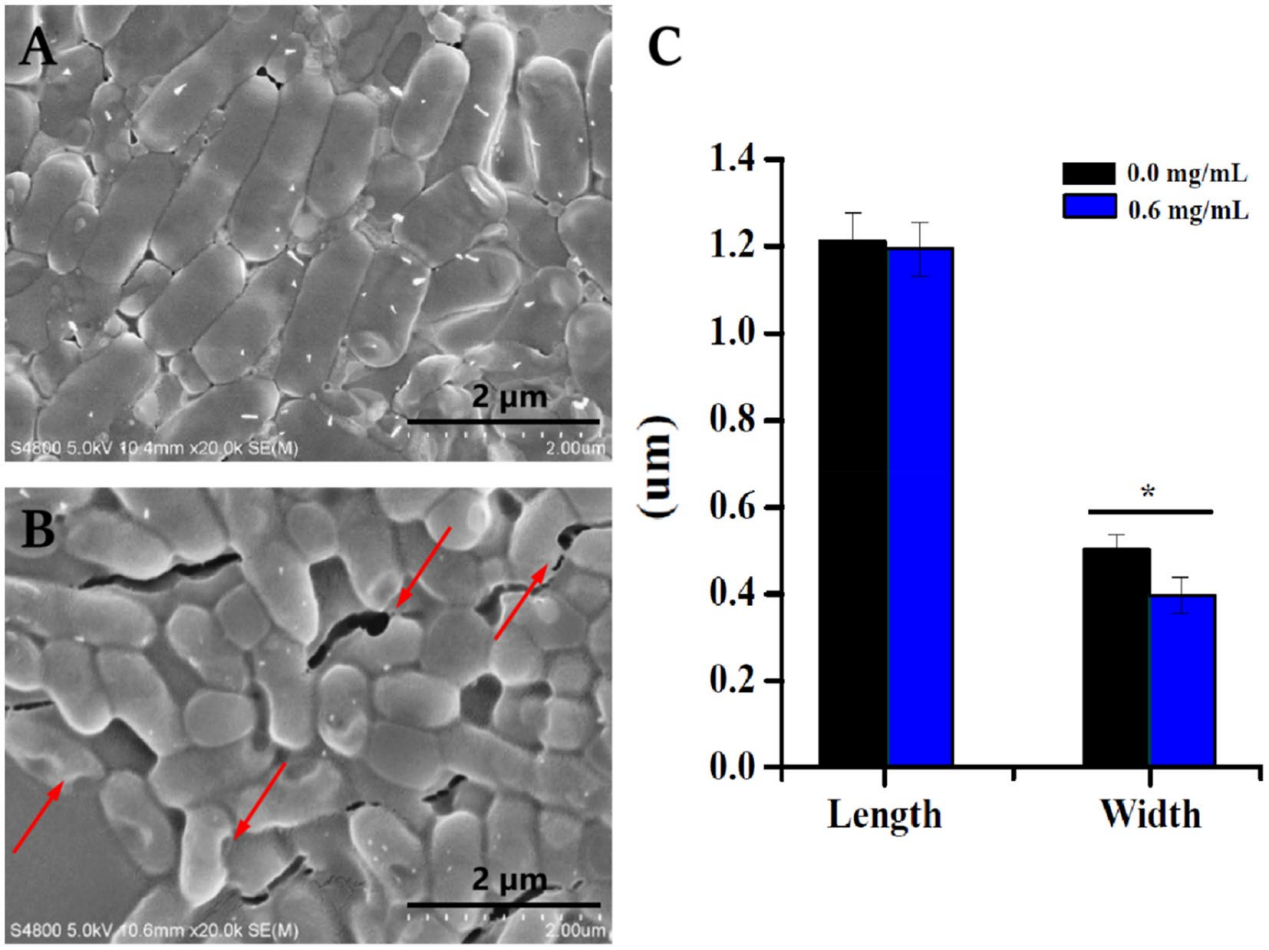
(**A**) Images of *R. solanacearum* cells obtained by scanning electron microscopy *R. solanacearum* untreated. (**B**) *R. solanacearum* treated with 0.6 mg/mL of NaHS, and (**C**) the effects of various treatments on the length and width of *R. solanacearum* cells. The red arrow denotes cell membrane damage. The mean value of length and breadth was derived using the scale bars of SEM images, and * denotes statistically significant differences as determined by *t-tests* with a *p*-value of < 0.05.

The cell morphology of *R. solanacearum* treated with 0.6 mg/mL NaHS was tracked using SEM to investigate the mechanism of antibacterial activity. Untreated *R. solanacearum* exhibited a long rod shape with a full membrane structure and a smooth texture (Fig. 2A). In contrast, NaHS treatment degraded the bacterial cells' surface structure and morphologies (Fig. 2B). *R. solanacearum* treated with 0.6 mg/mL NaHS had a shorter form than untreated *R. solanacearum*. In addition, treated *R. solanacearum* cells' diameters were much less than those of untreated *R. solanacearum* cells (Fig. 2C). These findings suggested that *R. solanacearum* morphology could be impacted by NaHS.

### Impact of NaHS on *R. solanacearum* biofilm formation

**Figure 3:**
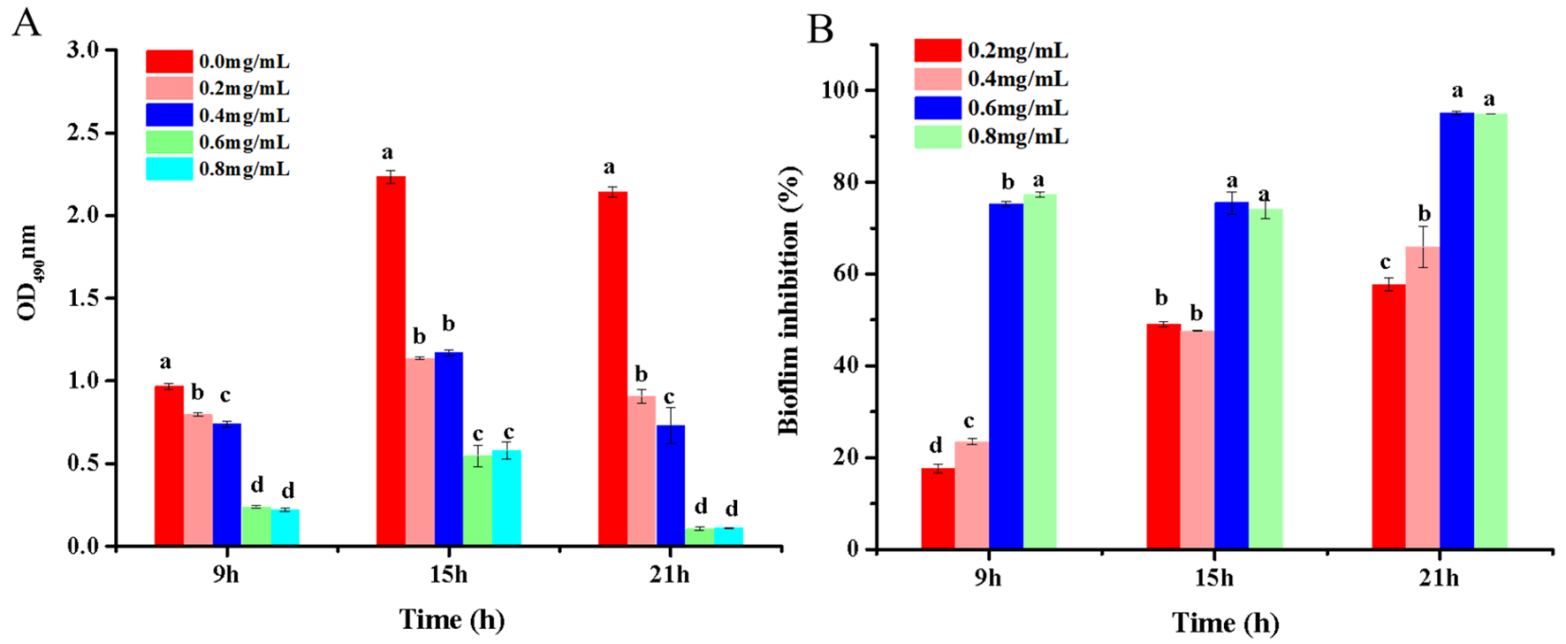
(**A**) The effects of various concentrations of NaHS on biofilm formation in *R. solanacearum*. (**B**) The inhibitory effect of NaHS on biofilm formation in *R. solanacearum*. The values are expressed as mean ± SE (n = 3). By Duncan's test, bars with lowercase letters indicate statistically significant differences (*p* < 0.05).

After 9, 15, and 21 h, the biofilm development of *R. solanacearum* was examined under 0.2, 0.4, 0.6, and 0.8 mg/mL concentrations of NaHS (Fig. 3). The biomass climbed from 9 to 15 h and dropped from 15 to 21 h in all treatments (Fig. 3A). Biofilm formation was substantially higher in the control (0.0 mg/mL) than in the other NaHS concentrations (0.2, 0.4, 0.6, and 0.8 mg/mL). Furthermore, treatments at 0.2 and 0.4 mg/mL could prevent biofilm formation, whereas treatments at 0.6 and 0.8 mg/mL substantially decrease biofilm formation. 0.6 and 0.8 mg/mL doses considerably lowered biofilm formation by 75.52% and 74.03% at 15 h and 95.05% and 94.82% at 21 h, respectively, compared to the control (Fig. 3B). All of these findings revealed that NaHS had a considerable inhibitory effect on *R. Solanacearum* biofilm development.

### Effects of NaHS on *R. solanacearum* swarming motility

**Figure 4:**
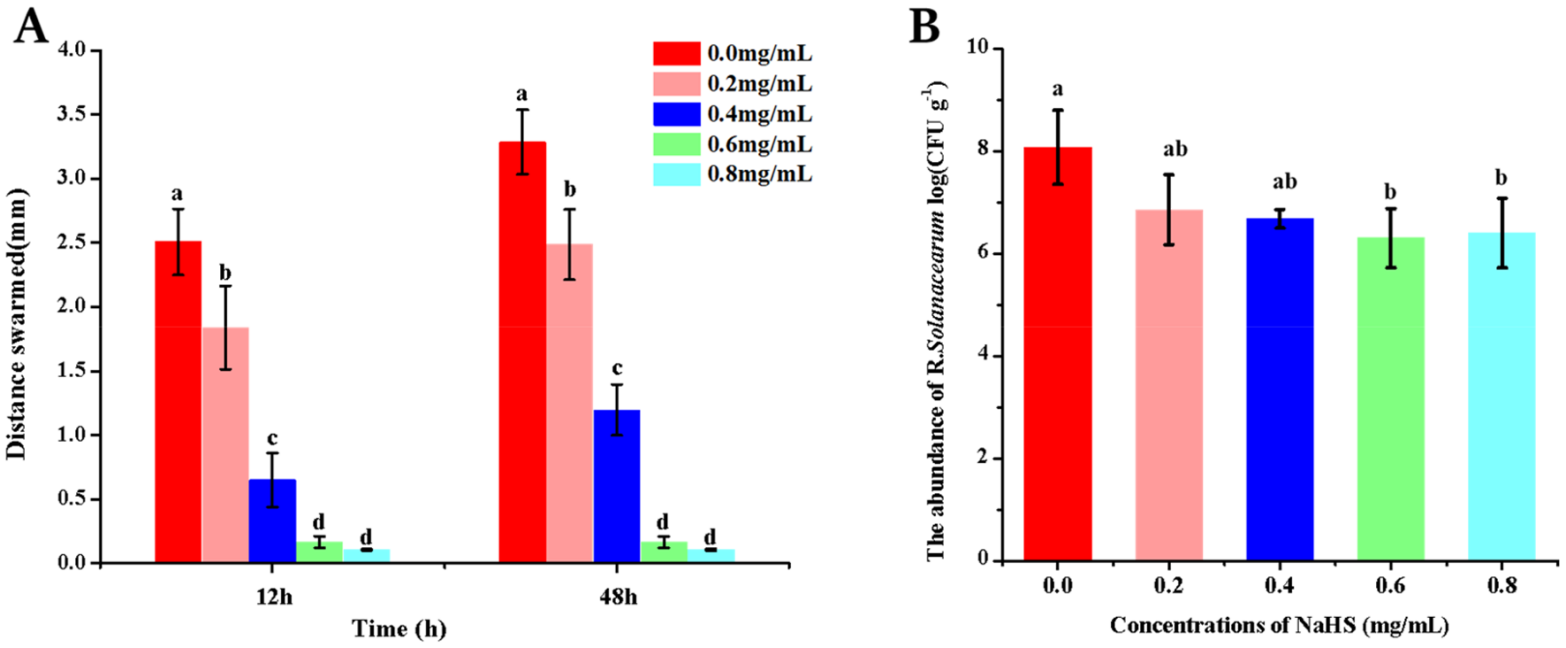
(**A**) The effects of varying concentrations of NaHS on the *swarming motility* of *R. solanacearum*. (**B**) The effects of different concentrations of NaHS on the colonization of tobacco roots by *R. solanacearum*. The values are presented as means ± SD (n = 3). In compliance with Tukey-HSD test, bars with lowercase letters indicate statistically significant differences (*p* < 0.05).

In Petri dishes, the effects of NaHS on *R. solanacearum* swarming motility were studied. At doses ranging from 0.2 to 0.8 mg/mL after 12 and 48 h, NaHS could substantially reduce the swarming motility of *R. solanacearum*, as presented in Fig. 4. The migratory zone's diameter gradually shrank as the concentration of NaHS raised. Furthermore, the diameter of the migration zone was significantly reduced by the 0.6 and 0.8 mg/mL treatments, although there was no noticeable difference between the 0.6 and 0.8 mg/mL treatments (Fig. 4A).

### Effect of NaHS on *R. solanacearum* colonization in tobacco roots

Based on the results of the experiments described above, NaHS could inhibit the growth of *R. solanacearum.* Thus, we subsequently evaluated the effect of NaHS on the colonization of *R. solanacearum* in tobacco roots under hydroponic conditions. As shown in Fig. 4B, NaHS significantly decreased the population of *R. solanacearum* that colonized the roots of tobacco plants at concentrations ranging from 0.2 to 0.8 mg/mL.

### Effects of NaHS on the transcriptome of *R. solanacearum*

**Figure 5:**
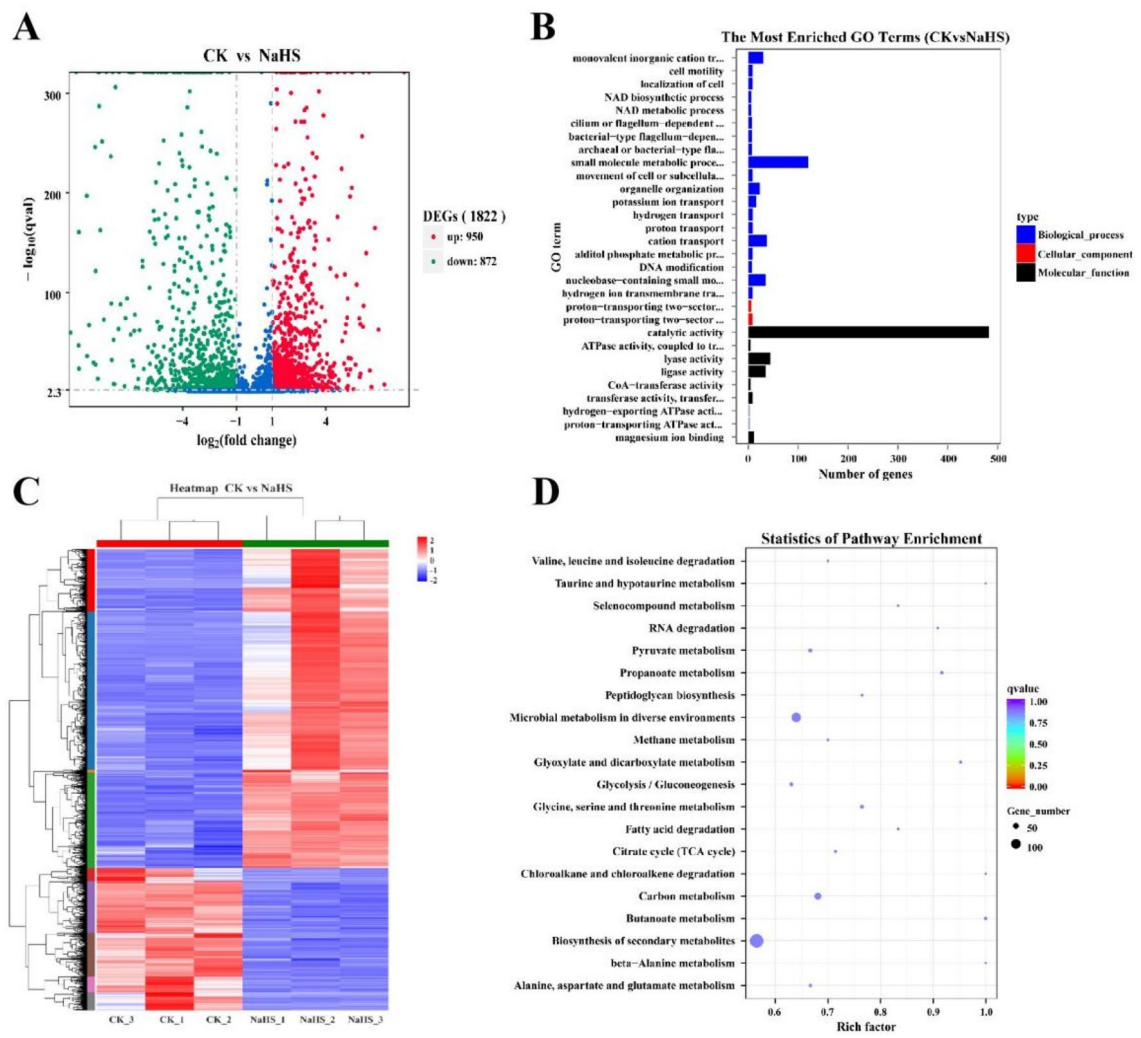
CK and NaHS transcriptomic analyses. (**A**) All identified genes volcano plot. (**B**) GO term enrichment analysis of DEGs. (**C**) DEG heatmaps. (**D**) KEGG pathway enrichment analysis of DEGs. CK represents untreated *R. solanacearum*, while NaHS denotes *R. solanacearum* treated with 0.6 mg/mL NaHS.

We examined and contrasted the transcriptomes of *R. solanacearum* treated with 0.0 mg/mL (the control) and 0.6 mg/mL NaHS to ascertain the impact of NaHS on the gene expression profile of *R. solanacearum*. Figure 5 illustrates how the NaHS treatment significantly changed *R.* *solanacearum* transcriptome compared to the control. A total of 1822 genes expressed differently, with 950 exhibiting increased expression and 872 demonstrating decreased expression (Fig. 5A). The DEGs were primarily dispersed in 3 categories, including biological processes, cellular components, and molecular activities, according to GO term enrichment analysis. The catalytic activity in molecular processes was enhanced. More enriched biological processes include motility, translation, transmembrane transport, and transport (Fig. 5B). The NaHS treatment group displayed distinct expression patterns compared to the control group (Fig. 5C). The top 20 metabolic pathways were enriched in KEGG analysis for metabolic pathways, microbial metabolism in various settings, carbon metabolism, biosynthesis of secondary metabolism, and biosynthesis (Fig. 5D). It was evident that *R. solanacearum* pathways had changed due to NaHS treatment based on the outcomes of the volcano plots, GO, and KEGG pathway analyses.

### Effects of NaHS on the incidence and index of TBW

According to the aforementioned experimental findings, NaHS can inhibit *R. solanacearum*. Thus, we conducted a pot experiment to establish the effects of NaHS on TBW. 10 d after inoculation, the control and NaHS treatments developed symptoms of bacterial wilt. The occurrence of TBW then increased more quickly in the control (CK) group than in the NaHS group (Table 1). When compared to 0.2 mg/mL and 0.4 mg/mL treatments from 18 to 20 days, 0.6 mg/mL and 0.8 mg/mL NaHS might considerably lower the disease incidence. The findings demonstrated that NaHS may substantially minimize the occurrence of TBW and that this incidence was considerably lower than that of the control (Table 1).

**Table 1:** The effect of NaHS on the occurrence of tobacco bacterial wilt in pot experiment.

Treatments	Disease incidence (10d)	Disease incidence (12d)	Disease incidence (14d)	Disease incidence (16d)	Disease incidence (18d)	Disease incidence (20d)
CK	7.68 ± 1.09 a	17.98 ± 3.41 a	42.14 ± 6.47 a	52.35 ± 8.94 a	61.85 ± 7.04 a	64.97 ± 9.46 a
NaHS200	4.36 ± 2.36 b	9.85 ± 0.31 b	17.69 ± 2.74 b	19.68 ± 5.81 b	28.19 ± 4.16 b	31.95 ± 3.75 b
NaHS400	3.12 ± 0.14 c	5.16 ± 0.38 c	10.23 ± 1.64 c	17.31 ± 3.47 b	19.64 ± 4.75 c	21.39 ± 2.64 c
NaHS600	2.16 ± 0.96 c	3.93 ± 1.02 c	5.46 ± 3.02 c	7.38 ± 1.63 c	8.64 ± 0.98 d	8.93 ± 1.69 d
NaHS800	2.07 ± 0.14 c	3.06 ± 0.84 c	4.21 ± 1.24 c	6.98 ± 0.21 c	8.04 ± 0.86 d	9.54 ± 1.01 d

All data are presented as the mean ± SE. The different lowcase letters in the same column indicate significant differences at *p* < 0.05 based on LSD test among different concentrations of NaHS.

Additionally, we assessed NaHS's ability to control the bacterial wilt-infected tobacco field. Disease incidence (I) and disease index (DI) was substantially greater in the control group than in the NaHS-treated group. As the concentration of NaHS increased from 0.2 to 0.8 mg/mL, the I continuously decreased from 44.22 to 15.21%, and the DI decreased from 13.21 to 4.26%. With the increase of NaHS concentration, the control efficacy increased gradually. All the results suggested that applying NaHS reduces the I and DI of BWT, and the control efficacy of TBW is as high as 89.49% (Table 2).

**Table 2:** The effect of NaHS on the occurrence of tobacco bacterial wilt in field experiment.

Treatments	Disease incidence (%)	Disease index	Control efficacy (%)
CK	89.34 ± 3.69 a	40.56 ± 2.29 a	0 c
NaHS200	44.22 ± 5.94 b	13.21 ± 1.52 b	67.46 ± 2.82 b
NaHS400	29.35 ± 6.99 c	8.76 ± 1.10 bc	78.41 ± 2.73 ab
NaHS600	20.58 ± 2.34 cd	6.93 ± 1.89 c	82.98 ± 3.32 a
NaHS800	15.21 ± 1.63 d	4.26 ± 1.07 cd	89.49 ± 1.23 a

All data are presented as the mean ± SE. The different lowcase letters in the same column indicate significant differences at *p* < 0.05 based on LSD test among different concentrations of NaHS.

### Effects of NaHS application on soil physicochemical properties

Seven physicochemical properties of the rhizosphere soil were analyzed (Table 3). The values of pH, alkali-hydrolyzed nitrogen (AN), available phosphorous (AP), and organic matter (OM) were increased as the concentration of NaHS increased from 0.2 to 0.8 mg/mL. There was no significant difference in available potassium (AK) and exchangeable calcium (Ca) between NaHS treatments and CK. The results showed that applying NaHS could increase the soil pH, AN, AP, and OM. What's more, pH, AN, AP, and OM showed a significantly negative (*P* < 0.01) correlation with the incidence of BWT (Supplementary Table S1). These results indicated that applying NaHS may reduce the incidence of BWT by changing soil physicochemical properties.

**Table 3:** The effects of different concentrations of NaHS on soil physicochemical properties.

Treatments	pH	AN (mg/kg)	AP (mg/kg)	AK (mg/kg)	OM (%)	Ca (mg/kg)	Mg (mg/kg)
CK	5.22 ± 0.13 d	141.58 ± 7.18 d	75.30 ± 3.60 c	770.44 ± 25.23 a	2.40 ± 0.19 d	999.04 ± 31.58 a	128.47 ± 36.52 a
NaHS200	5.59 ± 0.15 c	164.39 ± 3.80 c	84.00 ± 1.24 bc	756.87 ± 25.69 a	3.18 ± 0.10 c	987.96 ± 62.02 a	123.03 ± 52.82 a
NaHS400	6.14 ± 0.07 b	170.31 ± 3.44 bc	85.92 ± 4.31 bc	744.38 ± 36.12 a	3.38 ± 0.16 bc	990.50 ± 74.40 a	120.25 ± 28.00 a
NaHS600	6.36 ± 0.09 ab	184.48 ± 3.59 ab	97.52 ± 5.88 ab	750.69 ± 41.77 a	3.69 ± 0.07 b	998.58 ± 120.40 a	112.69 ± 18.00 a
NaHS800	6.49 ± 0.06 a	188.55 ± 6.98 a	115.75 ± 11.42 a	754.51 ± 8.87 a	4.19 ± 0.13 a	1005.04 ± 59.29 a	103.64 ± 39.15 a

Soil chemical properties in soils are presented as the mean ± SE. The different lowercase letters in the same column indicate significant differences at *p* < 0.05 based on Tukey-HSD test among different concentrations of NaHS.

### Effects of NaHS application on bacterial diversity and community

After quality filtering, a total of 679,451 high-quality raw sequences with an average length of 252 bps for bacteria were obtained from rhizospheric soil samples. The OTUs, Chao1, and Shannon index were used to evaluate and compare the richness and diversity of bacterial communities among different treatments (Supplementary Table S2). The OTUs, Chao1, and Shannon index in NaHS200 and CK treatments in the rhizosphere soil were insignificant. While the OTUs, Chao1, and Shannon index of NaHS400, NaHS600, and NaHS800 treatments were significantly lower than NaHS200 and CK treatments. With the increase of NaHS concentration from 0.2 to 0.6 mg/mL, the OTUs and Chao1 decreased gradually (Supplementary Table S2). This result suggested that applying NaHS could change the richness and diversity of the soil bacterial community.

**Figure 6:**
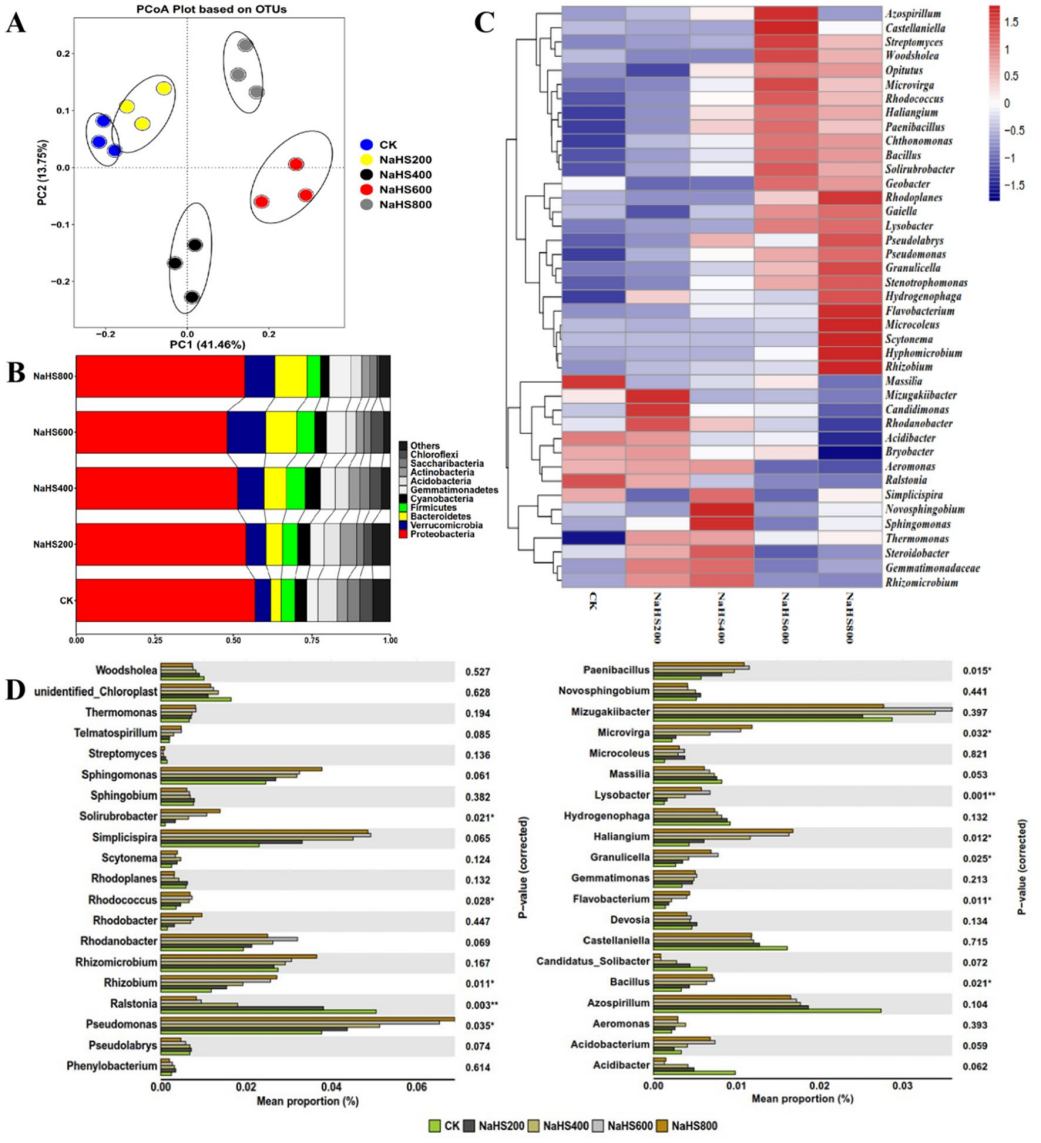
Bacterial community in the soil after five treatments. (**A**) The principal coordinate analysis (PCoA) of the soil bacterial community. (**B**) The relative abundance of bacterial phyla in soil samples. (**C**) A hierarchical cluster analysis of the top 40 classified bacterial genera and (**D**) The relative abundances of the top 40 classified bacterial genera among different treatments are all shown. The relative abundance of several taxa was examined using one-way ANOVA. *p < 0.05, **p < 0.01. (CK is 0.0 mg/mL NaHS, NaHS200 is 0.2 mg/mL NaHS, NaHS400 is 0.4 mg/mL NaHS, NaHS600 is 0.6 mg/mL NaHS, and NaHS800 is 0.8 mg/mL NaHS).

Principal coordinate analysis (PCoA) was carried out using weighted UniFrac distance in different treatments, and PC1 and PC2 explained 55.21% of the total bacterial community. Bacterial communities from CK and NaHS200 were clustered together. In contrast, the CK, NaHS400, NaHS600, and NaHS800 were separated (Fig. 6A). This result indicated that NaHS400, NaHS600, and NaHS800 treatments' bacterial community structure was different from CK. A total of 50 bacterial phyla were identified from all soil samples. The top ten abundant bacterial phyla were selected to compare the five treatments' bacterial community changes in rhizosphere soil. The relative abundance of the top ten predominant phyla totaled up to 94.27–97.87% (Fig. 6B). Among the top ten bacterial phyla, *Proteobacteria* included the pathogen *R. solanacearum* was the most dominant (48.01–56.91%), and followed by *Verrucomicrobia* (5.02–12.25%), *Bacteroidetes* (3.28–10.33%), *Firmicutes* (4.24–5.92%), *Gemmatimonadetes* (3.55–6.94%), *Acidobacteria* (3.24–6.13%), *Actinobacteria* (2.24–5.28%), *Saccharibacteria* (2.24–3.84%), *Cyanobacteria* (2.74–4.89%) and *Chloroflexi* (0.92–4.23%). The relative abundance of *Proteobacteria* in NaHS800 treatment was lower than in other treatments. In comparison, the relative abundance of *Verrucomicrobia* and *Bacteroidetes* was higher than that in other treatments (Fig. 6B). The heatmap analysis of the top 40 genera with hierarchical clusters was used to identify the different compositions of bacterial community structure. There were distinctions in bacterial community structures among different treatments in the heatmap. Compared to the CK, the relative abundances of *Streptomyces*, *Woodsholea, Microvirga*, *Rhodococcus*, *Haliangium*, *Chthonomonas*, *Bacillus*, *Solirubrobacter*, *Geobacter, Rhodoplanes, Gaiella*, *Lysobacter*, *Pseudomonas*, *Granulicella*, *Stenotrophomonas*, *Flavobacterium* and *Rhizobium in* NaHS600 and NaHS800 treatments significantly increased. (Fig. 6C). The application of NaHS significantly decreased the relative abundances of *Massilia*, *Acidibacter,* and *Ralstonia* (pathogen of bacterial wilt). These results suggested that NaHS application influences the structure of the bacterial community.

Further analyses were carried out at the genus level. The different distributions of the top forty abundant bacterial genera among the five treatments were illustrated in Fig. 6. Twelve varied among the five treatments were significantly different, including *Solirubrobacter*, *Rhodococcus*, *Rhizobium*, *Ralstonia*, *Pseudomonas*, *Paenibacillus*, *Microvirga*, *Lysobacter*, *Haliangium*, *Granulicella*, *Flavobacterium*, and *Bacillus*. The genus *Solirubrobacter*, *Rhodococcus*, *Rhizobium*, *Pseudomonas*, *Paenibacillus*, *Microvirga*, *Lysobacter*, *Haliangium*, *Granulicella*, *Flavobacterium*, and *Bacillus* were dominant in NaHS treatments and occupied a low percentage in CK (Fig. 6D). In contrast, *Ralstonia* was dominant in CK and decreased significantly in NaHS treatments (Fig. 6D).

### Effects of NaHS application on fungal diversity and community

The difference between the OTUs, Chao1, and Shannon fungal community index among different treatments was also analyzed (Supplementary Table S2). There was no significant difference in OTUs, Shannon, and Chao1 indexes between CK and NaHS 200. With an increase in NaHS concentration from 0.4 to 0.8 mg/mL, the OTUs, Chao1, and Shannon index were reduced significantly. The results showed that NaHS application could impact the diversity and richness of soil fungi.

**Figure 7:**
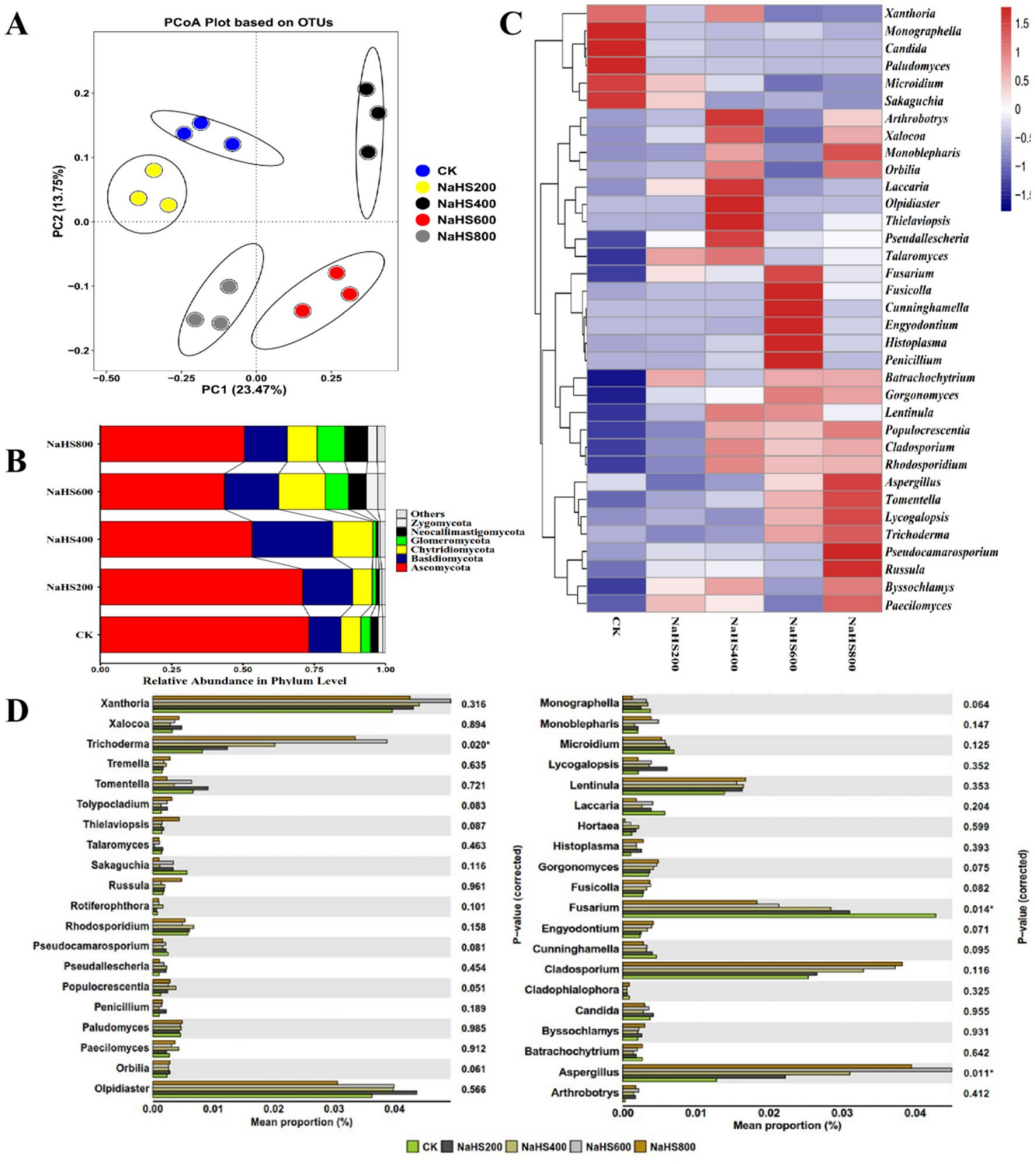
Soil fungi in five different treatments. (**A**) The relative abundance of bacterial phyla in soil samples. (**B**) The principal coordinate analysis (PCoA) of the soil fungal community. (**C**) The hierarchical cluster analysis of the most common fungal genera and (**D**) the relative abundances of the top 40 classified fungal genera among various treatments. Different genera abundances were examined using a one-way ANOVA. *p < 0.05, **p < 0.01. (CK: 0.0 mg/mL NaHS, 0.2 mg/mL NaHS, 0.4 mg/mL NaHS, 0.6 mg/mL NaHS, and 0.8 mg/mL NaHS for NaHS200, 400, 600, and 800).

According to PCoA analysis, PC1 and PC2 explained 23.47% and 13.75% of the total fungal community variations, respectively (Fig. 7A). The distribution of fungi among different treatments was relatively discrete, indicating obvious differences in the fungal community structure among different treatments. Top six fungal phyla were identified from all soil samples, including *Ascomycota* (43.56–73.45%), followed by *Basidiomycota* (7.98–28.21%), *Chytridiomycota* (6.84–30.15%), *Glomeromycota* (1.00–8.16%), *Neocallimastigomycota* (0.05–6.19%) and *Zygomycota* (0.66–9.31%) (Fig. 7B). The relative abundance of *Ascomycota* decreased as the concentration of NaHS increased from 200 to 600 mg/L. The relative abundance of *Basidiomycota*, *Glomeromycota*, *Chytridiomycota*, and *Zygomycota* also varies with the concentration of NaHS. These results indicated that NaHS altered the fungal community composition associated with NaHS concentration. In the heatmap for the fungal community, the relative abundance of *Monograpella*, *Candida*, *Paludomyces*, *Microidium*, and *Sakaguchia* in CK was significantly higher than in NaHS treatment. NaHS800 significantly enriched the relative abundance of *Batrachochytrium*, *Gorgonomyces*, *Populocrescentia*, *Cladosporium*, *Rhodosporidium*, *Aspergillus*, *Tomentella*, *Lycogalopsis*, *Trichoderma*, *Pseudocamarosporium*, *Russula*, *Byssochlamys*, and *Paecilomyces* (Fig. 7C).

The distributions of the top forty abundant fungi at the genus level among the five treatments were analyzed (Fig. 7D). Three were significantly different among the five treatments, including *Trichoderma*, *Fusarium*, and *Aspergillus*. Genus *Trichoderma* and *Aspergillus* were dominant in NaHS and occupied a low percentage in CK (Fig. 7D). In contrast, *Fusarium* was dominant in CK and decreased significantly in NaHS treatments (Fig. 7D).

### Relationship between rhizosphere soil physicochemical properties and microbial community

**Figure 8:**
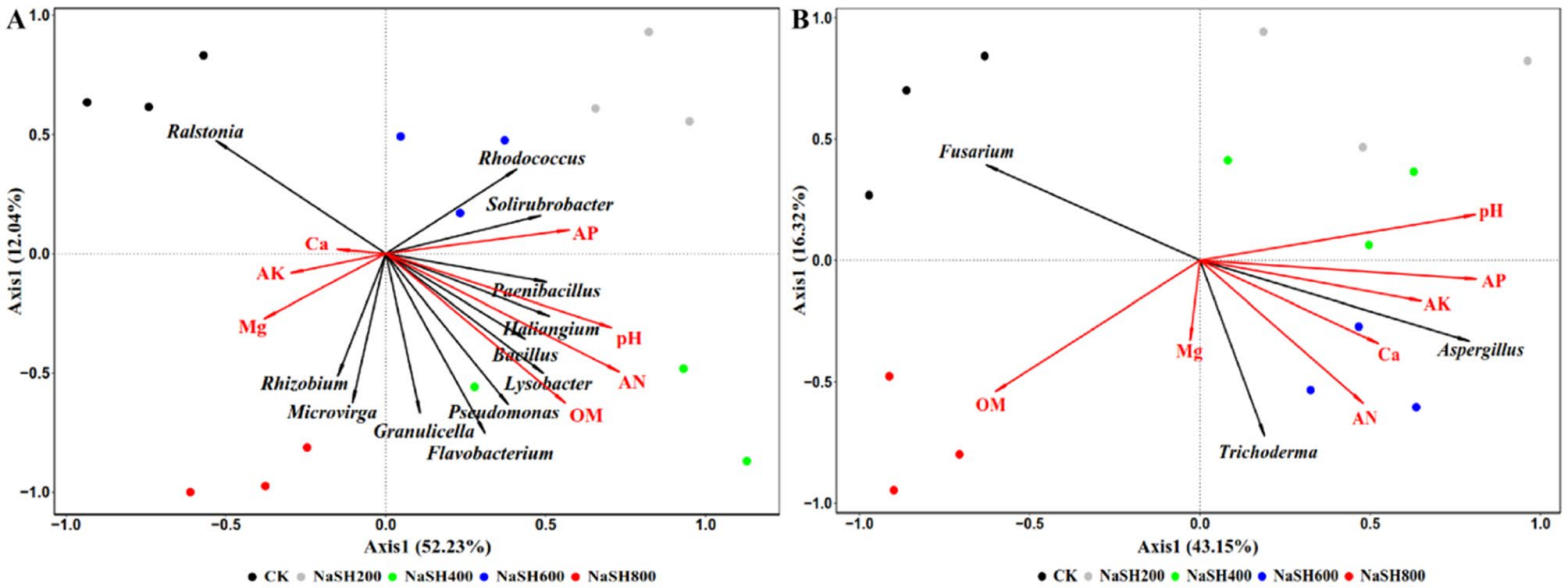
Analysis of duplication between soil physicochemical characteristics and rhizosphere microbial community. (**A**) Bacterial and (**B**) fungal communities. (CK is 0.0 mg/mL NaHS, NaHS200 is 0.2 mg/mL NaHS, NaHS400 is 0.4 mg/mL NaHS, NaHS600 is 0.6 mg/mL NaHS, and NaHS800 is 0.8 mg/mL NaHS).

The relationship between rhizosphere soil physicochemical properties and microbial community structure was analyzed by redundancy analysis (RDA). The results showed 64.27% and 59.47% of bacterial and fungal community variation, respectively (Fig. 8). The bacterial *Rhodococcus*, *Solirubrobacter*, *Paenibacillus*, and *Haliangium*, *Bacillus*, *Lysobacter*, *Pseudomonas*, *Flavobacterium*, and *Granulicella* were positively correlated with pH, AN, AP, and OM. While, *Ralstonia* presented contrasting behavior negatively associated with pH, AN, AP, and OM, as shown in Fig. 8A. *Trichoderma* and *Aspergillus* were positively linked with AN, Ca, AK, AP, and pH. On the other hand, *Fusarium* negatively correlated with AN, Ca, AK, AP, and pH (Fig. 8B). The redundancy analysis revealed that rhizosphere soil AN, Ca, AK, AP, and pH greatly influenced the microbial community.

## Discussion

In this study. We determined that NaHS greatly decreased *R. solanacearum* growth and affected its morphology, biofilm, and transcriptome (Figs. 1, 2, 3, 4, 5). Furthermore, NaHS treatment might regulate TBW (Tables 1, 2), change soil physicochemical parameters (Table 3), and regulate the soil microbial community (Figs. 6, 7, 8).

H_2_S possesses several biological activities, including cell signaling and antibacterial and antifungal properties^23,24,26^. Our findings demonstrated that NaHS is a powerful antibacterial agent against *R. solanacearum* (Fig. 1). According to the SEM results, NaHS may disrupt the cell morphology, resulting in the death of *R. solanacearum* (Fig. 2). As per earlier research, several antibacterial agents target the cell membrane first and then inhibit *R. solanacearum* by destroying the cell membrane^21,27^. Furthermore, we observed that NaHS prevented *R. solanacearum* biofilm formation, swarming motility, and colonization ability (Figs. 3, 4), similar to prior work^28^. We investigated the transcriptome of *R. solanacearum* better to study the antibacterial mechanism of NaHS against *R. solanacearum*. In NaHS-treated *R. solanacearum*, 1822 genes are engaged, with 950 up-regulated and 872 down-regulated (Fig. 6). The transcriptome of *R. solanacearum* is dramatically altered by NaHS treatment. These data suggested that NaHS are involved in antibacterial activity.

Antibacterial drugs are an effective technique to control pathogens. Lysine had a controlled efficacy of 58–100% on tomato bacterial wilt in pot experiments^29^. Hydroxycoumarins can potentially reduce TBW by 38.27–80.03%^30^. In this investigation, we discovered that using NaHS may effectively prevent TBW, with control efficacy reaching 89.49% in the field experiment (Table 2). According to the findings, NaHS could be employed as an antibacterial agent to control TBW.

Since researchers indicated that the exogenous application of H_2_S can affect plant growth by altering soil nutrient content^31,32^, in our study, the application of NaHS also increased soil pH, AN, AP, and OM values (Table 3). Increased pH is important for inhibiting the survival of *R. solanacearum* and increasing OM, N, and P to meet the needs of plant growth^33,34^. In addition, the higher soil phosphorus could increase the activity of beneficial microorganisms against the pathogen^34^. This study demonstrated that the application of NaHS significantly reduced the disease incidence and disease index of TBW, and pH, AN, AP, and OM showed a significantly negative correlation with the incidence of TBW (Table 2, Supplementary Table S1). It was hypothesized that administering NaHS could also modify the physicochemical features of the soil.

The rhizosphere soil microbial community structure influences the plant's immunity and quality, and the microbial community is considered a key mechanism that can suppress soil-borne pathogens^35,36^. Our findings explored that applying NaHS altered the diversity and richness of soil microbes (Supplementary Table S2), which was consistent with the results of Fang et al.^32^. The microbial analysis revealed a different treatment pattern (Figs. 6A, 7A). NaHS application significantly influenced the composition of the soil microbial community (Figs. 6, 7). NaHS treatments reduced the relative abundance of *Proteobacteria* (Fig. 6B), which was similar to the results by Li et al.^35^. The phylum *Proteobacteria* including the pathogen *R. solanacearum,* is less abundant in healthy soils than in bacterial wilt soil^35^. NaHS treatments also reduced the relative abundance of *Acidobacteria* (Fig. 6B). *Acidobacteria* was mainly driven by soil pH, and the low pH was more suitable for the survival of *Acidobacteria*^37^. Our results also showed that the relative abundance of *Bacteroidetes* was increased in NaHS treatments (Fig. 6B), which could promote plant growth and improve the resistance of plants to environmental stress^38^. The heatmap based on significant changes indicated that NaHS application increased the abundances of some bacteria and fungi (such as *Paenibacillus*, *Bacillus*, *Lysobacter*, *Aspergillus,* and *Trichoderma,* etc). (Figs. 6C, 7C). These microorganisms are recognized as beneficial microorganisms, and their functions may be related to soil physicochemical properties, accelerating the cycling of elements, promoting plant growth, and environmental adaption^39–42^.

In the present investigation, specific genera of microorganisms of S*olirubrobacter*, *Rhodococcus*, *Rhizobium*, *Pseudomonas*, *Paenibacillus*, *Microvirga*, *Lysobacter*, *Haliangium*, *Granulicella*, *Flavobacterium*, *Bacillus*, *Trichoderma*, and *Aspergillus* were significantly high in NaHS treatments. However, *Ralstonia* and *Fusarium* were significantly low in NaHS treatments (Figs. 6D, 7D). The genus *Ralstonia* includes many soil-borne pathogens, which only infect roots via wounds caused by microbes and insects^43^. Pathogenic *Fusarium* can infect the root by penetrating hyphae, causing more wounds to the root, thus increasing the root infection by pathogenic *Ralstonia*^44^. The application of NaHS could inhibit bacterial wilts caused by pathogenic *Ralstonia.* Some species of *Solirubrobacter* and *Granulicella* positively affect the soil's organic carbon transition^45^. *Rhodococcus* and *Bacillus* were reported as phosphate-mobilizing bacteria, which can solubilize organic and inorganic phosphate^46^. Furthermore, some species of the genus *Bacillus* can affect the growth and virulence traits of *Ralstonia* by producing volatile organic compounds^41^. *Pseudomonas* promotes plant growth, inhibits pathogens, and induces systemic resistance to diseases in many plants^47^. *Microvirga* is a nitrogen-fixing bacteria that can be involved in nitrogen cycling^46^. Previous studies have documented that *Pseudomonas*, *Rhizobium*, *Paenibacillus*, *Lysobacter*, *Haliangium*, *Flavobacterium*, and *Bacillus,* as antagonistic bacteria, can mitigate many soil-borne diseases and promote plant growth and health^40,41,48,49^. *Trichoderma* and *Aspergillus* have been reported as antagonistic fungi. They directly interact with roots to produce bioactive substances, improve plant growth, and resist abiotic and biotic stress^40,42^. The application of NaHS may provide a suitable environment for promoting the growth of these beneficial microorganisms, increasing the relative abundance of these beneficial microorganisms, and reducing the incidence of BWT. The redundancy analysis (RDA) revealed that these beneficial microorganisms were positively correlated with pH, AN, and AP, whereas *Ralstonia* was negatively correlated with pH, AN, and AP (Fig. 8).

In this study, we revealed that NaHS considerably reduced the growth of *R. solanacearum* in the root by controlling the root's biofilm production, transcriptome, and colonization. Similarly, the occurrence and disease index of TBW decreased dramatically through using NaHS. Moreover, the exogenous application of NaHS can affect the soil's physiochemical properties and influence the constitution and arrangement of the soil's microbial population, contributing to the management of TBW. These results might offer empirical and theoretical justification for NaHS-mediated TBW control. Future studies are expected to evaluated the effect of NaHS on the expression of biofilm formation-related genes and virulence-associated genes of *R. solanacearum.*

## Materials and methods

### Tobacco materials, bacterial strains, culture conditions, and reagents

The Tobacco Research Institute of Hubei in Wuhan, China, provided the Yunyan87 for this study. The *R. solanacearum* (phylotype I, race 1, biovar III) was used in this study^50^. *R.* *solanacearum* was routinely grown on a semisolid medium containing 0.35% agar, nutrient broth medium (NB), and nutrient agar (NA) medium. For hydroponic tobacco, Murashige and Skoog (MS) media was utilized. NaHS was acquired from Aladdin Reagent Co., Ltd. (Shanghai, China) for use in this study.

### Evaluating NaHS antibacterial activity against *R. solanacearum* in vitro

Agar dilution assays and growth curves at various doses were used to assess the influence of NaHS on the growth of *R. solanacearum*. 100 µL of newly developed *R. solanacearum* suspension (1 × 10^9^ CFU/mL) was disseminated directly onto NA plates with varying concentrations (0.0, 0.2, 0.4, 0.6, 0.8, and 1.0 mg/mL) of NaHS. The plates were incubated at 28 ± 1 °C for 24 h to count the colonies. The viability of the *R. solanacearum* cell was then measured by counting CFU on the agar plates. Cell viability (%) = V′/V × 100% formula was used to calculate the viability of the cells, where V′ and V stand for the number of colony-forming units on the control plates (0.0 mg/mL NaHS) and NaHS (0.2, 0.4, 0.6, 0.8, and 1.0 mg/mL) plates, respectively.

The *R. solanacearum* was freshly cultured in a suspension, and 100 µL of that suspension (1 × 10^9^ CFU/mL) was transferred to NB medium with various NaHS concentrations (0.0, 0.2, 0.4, 0.6, 0.8, and 1.0 mg/mL). The cultures were incubated at 30 °C for 36 h while shaken at 180 rpm. The optical density of 600 nm (OD_600_) was measured every 3 h using a Nicolet Evolution 300 UV–Vis spectrometer to obtain the growth curves. Each treatment was carried out three times and calculated to obtain an average value.

### The influence of NaHS on the morphology of *R. solanacearum*

A scanning electron microscope (SEM) was used to examine the morphology of *R. solanacearum* cells in the presence of NaHS. *R. solanacearum* was diluted in NB medium to 1 × 10^9^ CFU/mL solution. NaHS was added to the suspension of *R. solanacearum* at a final concentration of 0.6 mg/mL. The medium devoid of NaHS served as the control. The cultures were incubated at 30 °C for 12 h with 180 rpm shaking. After 12 h, the cells of *R. solanacearum* were removed by centrifugation at 6000 rpm for 5 min, washed three times with 0.1 mol/L pH 7.0 phosphate buffer, and fixed with 2.5% of glutaraldehyde overnight at 4 °C. The SEM analysis was performed as described by Li et al.^21^.

### Effect of NaHS on biofilm formation of *R. solanacearum*

*Ralstonia solanacearum* biofilm formation was assessed using crystal violet staining, as outlined by Peeters et al.^51^, with minor modifications. The experiment was carried out in 96-well microtiter plates. To induce biofilm growth, 100 L of mixed cultures (10 µL of inoculum (OD_600_ ≈ 1.0) mixed with a final NaHS concentration of 0, 0.2, 0.4, 0.6, and 0.8 mg/mL in NB medium) were inoculated into individual wells of a 96-well microtiter plate. The plates were wrapped with cling wrap and incubated at 30 °C without shaking for 9, 15, and 21 h before the liquid medium was withdrawn, and the plates were rinsed three times with distilled water. Each well was stained for 30 min with 0.1% crystal violet, followed by three washes with water to eliminate any remaining stain. 200 µL of 95% ethanol was used to remove the crystal violet, and then a microplate reader was used to measure the biofilm's absorbance at 490 nm. The experiment was carried out in triplicate, with each treatment repeated three times.

### Effect of NaHS on swarming motility of *R. solanacearum*

With slight modifications, the swarming assay was based on Tans-Kersten et al.^52^. study. In Petri dishes, a semisolid medium containing 0.35% agar and NaHS at various doses (0, 0.2, 0.4, 0.6, and 0.8 mg/mL) was added before being air-dried. The OD_600_ ≈ 0.8 *R. solanacearum* overnight culture was collected, twice washed in sterile water at 6000 rpm for five minutes, and then resuspended in sterile water. Drop-inoculated 2 µL of *R. solanacearum* suspension onto the center of plates. They were then incubated at 30 °C for 12 and 48 h. On each plate, the colony diameters were measured in both the vertical and horizontal directions. Each assay was carried out three times. The average of three separate experiments was used to express the outcomes.

### Evaluation of *R. solanacearum* colonization in tobacco roots

A hydroponic experiment and plate counts were used to assess *R. solanacearum* colonization. Tobacco seedlings were rinsed three times with distilled water after reaching the 4–5 leaf stage, and tobacco roots were immersed in different concentrations of NaHS (0.0, 0.2, 0.4, 0.6, 0.8, and 1.0 mg/mL) for 30 min. The treated tobacco seedlings were grown in MS medium with 1 mL of *R. solanacearum* (1 × 10^9^ CFU/mL) added as an inoculant. The population of *R. solanacearum* adhering to the tobacco root was identified after 3 days of incubation in a greenhouse at 28 ± 1 °C with 85–90% relative humidity. The tobacco roots were cut out, submerged in 75% ethanol for 15 min, and then rinsed in sterile water. Each treated 1 g root was blotted dry before being pulverized in a mortar with 5 mL sterile water until finely homogenized. 1 mL of the supernatant was then collected as the bacterial suspension in the plant roots. The suspension was then sub-cultured and evenly placed onto NA medium before being maintained for 48 h at 30 °C. To determine the amount of *R. solanacearum* in tobacco roots per unit weight, the strains were then characterized, and the colonies were counted. At least three times were given for each treatment independently.

### Transcriptome analysis

Using a mirVana miRNA isolation kit (Ambion, Austin, TX, USA), total RNA from *R. solanacearum* treated with 0.0 (the control) and 0.6 mg/mL NaHS was extracted as recommended by the manufacturer. Using a Nano 6000 Assay Kit from the Bioanalyzer 2100 system (Agilent Technologies, CA, USA), the concentration and quality of the RNA were evaluated. Consequently, NEBNext^®^ UltraTM Directional RNA Library Prep Kit for Illumina^®^ (NEB, USA) was used to prepare 3 µg of total RNA from each sample following the manufacturer's instructions. The samples were sequenced on the Agilent Bioanalyzer 2100 system after synthesizing the first and second strand complementary cDNA. The readings were approximately 150 bp in length. FastQC was used to check the quality of the raw RNA-Seq data^53^. The *R. solanacearum* reference genome (NZ CP016914.1) was obtained from GeneBank, and Bowtie2-2.2.3 was used to align the reads to the reference genome sequences^54^. HTSeq v0.6.1 was used to count the number of reads aligned to each gene, and the FPKM of the genes was calculated using both gene lengths and the number of reads mapped^55^. The DESeq R package (1.18.0) was used to identify differentially expressed genes (DEGs). Gene Ontology (GO) was confirmed to be considerably enriched at p < 0.05 using the GOseq program in R^56^. Using KOBAS software (KOBAS, Surrey, UK), the Kyoto Encyclopedia of Genes and Genomes (KEGG) pathway analysis was performed, and the pathway with a corrected p < 0.05 was regarded as significantly enriched in DEGs^57^. Transcriptome analysis was carried out using 2 samples for each of the 3 biological replications per sample.

### Indoor pot and field experiments

An indoor pot experiment in the greenhouse was conducted to assess the control efficacy of NaHS on TBW. When tobacco seedlings reached the 4–5 leaf stage, the rhizosphere was infected with 1 mL of fresh *R. solanacearum* suspension (1 × 10^9^ CFU/mL). Two days after *R. solanacearum* inoculation, 10 mL of NaHS in various doses (0.0, 0.2, 0.4, 0.6, 0.8, and 1.0 mg/mL) was irrigated onto the tobacco roots. Tobacco seedlings injected with bacteria were grown in a greenhouse at 28 ± 1 °C and 85–90% relative humidity. Each treatment had three replicates, each one with 30 plants. Every 2 days, the disease's progression was recorded.

The field experiment was performed in tobacco fields with 15 years of continuous cropping tobacco fields in Xuan'en County (109°26′ E, 29°59′ N), Enshi City, Hubei province, China, from April to September 2019. TBW incidence has been higher than 95% yearly for the past 5 years^58^ when tobacco seedlings with 4–5 leaves were transplanted into the field. The experimental design consisted of three blocks, each 200 m^2^ in size, and each block was divided into five plots of 40 m^2^, with 60 plants in each plot. Five treatments with three replicates in completely randomized blocks were established. The treatments were: (1) control, 0.0 mg/mL NaHS (CK); (2) 50 mL, 0.2 mg/mL NaHS (NaHS200); (3) 50 mL, 0.4 mg/mL NaHS (NaHS400); (4) 50 mL, 0.6 mg/mL NaHS (NaHS600); (5) 50 mL, 0.8 mg/L NaHS (NaHS800). NaHS was applied to each tobacco root during transplantation. The planting density of all treatments was the same. TBW symptoms were tracked from 30 to 100 days after transplantation.

A prior study described the TBW disease index (DI) based on a severity scale of 0–9^21^. The TBW disease incidence (I) and disease index (DI) was calculated as follows: I = n/N × 100%, and DI = ∑(r × n′)/(N × 9) × 100, where "n" represents the total number of infected tobacco plants and "N" refers to the total number of plants, "r" represents the disease severity rating scale, and "n′" is the number of infected tobacco plants with a rating of r. Control efficiency = [(I of control − I of treatment)/I of control] × 100%.

### Soil sample collection and physicochemical properties analysis of rhizosphere

Rhizosphere soils were collected by the five-spot-sampling method at 50 d, 70 d, and 90 d post-transplantation^58^. Then the soil samples from the five separate sites were mixed into one soil sample and partitioned into two subsamples, one was immediately transported on ice to the laboratory and stored at − 80 °C for genomic DNA extraction, and the other subsamples were air-dried for physicochemical properties analysis. The analysis of soil pH, alkali-hydrolyzed nitrogen (AN), available phosphorus (AP), available potassium (AK), organic matter (OM), exchangeable calcium (Ca), and exchangeable magnesium (Mg) was performed according to Hu et al.^59^.

### Rhizosphere soil microbial community analysis

DNA was extracted from 0.5 g rhizosphere soil using the FastDNA Spin Kit (MP Biomedicals, USA) following the manufacturer's protocol. The integrity of DNA samples was determined by 1% agarose gel electrophoresis. Then the concentration and purity of the DNA were determined using a Nanodrop 1000 spectrophotometer (Nanodrop Technologies, Wilmington, DE, USA).

The extracted DNA from each soil sample was used as a template for amplification. The V4 region of the bacterial 16S rRNA genes and the ITS1 regions of the fungal rRNA genes were amplified. Each DNA sample was amplified separately using the following primers: Forward, 515F; (5′-GTGCCAGCMGCCGCGGTAA-3′), and Reverse, 806R; (5′-GGACTACHVGGGTWTCTAAT-3′) for bacterial^60^. Forward, ITS5-1737F; (5′-GGAAGTAAAAGTCGTAACAAGG-3′) and Reverse, ITS2-2043R; (5′-GCTGCGTTCTTCATCGATGC-3′) for fungi^61^. All PCR reactions were performed on Illumina HiSeq platforms (Illumina Inc., USA) at Novogene Bioinformatics Technology Co., Ltd (Beijing, China). The library quality was assessed on the Qubit@ 2.0 Fluorometer (Thermo Scientific) and the Agilent Bioanalyzer 2100 system. CASAVA1.8 statistically analyzed the sequence quality. The raw sequence data was filtrated using the FASTX Toolkit 0.0.13 software package. That removed the low mass base at the tail of the sequence (Q value < 20) and the sequences with lengths < 35 bp. Finally, the obtained size of the amplicon reads was approximately 250 bp. All effective tags of all samples were clustered using Uparse software (V7.0.1001, http://drive5.com/uparse/). Sequences with ≥ 99.5% identity for 16S rDNA and sequences with ≥ 97% identity for ITS were assigned to the same OTUs (operational taxonomic units). The OTUs, Chao1, and Shannon index were calculated with QIIME (Version 1.7.0) to evaluate the richness and diversity of soil microbial community^62^.

### Statistical analysis

One-way analysis of variance (ANOVA), the least significant difference (LSD) test, Tukey-HSD test, Duncan's test, and independent-sample *t*-tests with *p* values of < 0.05 and < 0.01 were used to analyze the data among the treatments. The statistical evaluations utilized SPSS version 18.0 (IBM, United States). The correlation analysis was conducted using the Pearson 2-tailed correlation test. Principal coordinate analysis (PCoA) with the weighted Unifrac distance and redundancy analysis (RDA) was carried out using R (Version 2.15.3).

## Supplementary Information

Supplementary Information.

## Data Availability

The datasets used and/or analysed during the current study are available from the corresponding author on reasonable request.
