# Supplementary material for: Effect and mechanism of NaHS on tobacco bacterial wilt caused by Ralstonia solanacearum
Source: Sci Rep. 2023 Feb 11;13:2462. doi: 10.1038/s41598-022-26697-8 (PMC9922318; doi:10.1038/s41598-022-26697-8)
Supplement: Supplementary file 1 — Supplementary Information. [file 41598_2022_26697_MOESM1_ESM.docx]

Supporting information

Effect and Mechanism of NaHS on tobacco bacterial wilt caused by *Ralstonia solanacearum*

Dingxin Wen^1a^, Qingqing Guo^1a^, Wan Zhao^1^, Yong Yang^1^, Chunlei Yang^2*^, Jun Yu^2*^, Yun Hu^1*^

^1^State Key Laboratory of Biocatalysis and Enzyme Engineering, College of life science, Hubei University, Wuhan 430062, China.

^2^Tobacco Research Institute of Hubei Province, Hubei Wuhan 430030,China

^a^ These authors have contributed equally to this work and share first authorship.

**^*^**E-mails: [huyun@hubu.edu;](mailto:huyun@hubu.edu;) [yujun80324@163.com;](mailto:yujun80324@163.com;) ycl193737@163.com.

**Table S1** Pearson correlation analysis between disease incidence of tobacco bacterial wilt and soil physicochemical properties.

|  | **pH** | **AN** | **AP** | **AK** | **OM** | **Ca^2+^** | **Mg^2+^** |
| --- | --- | --- | --- | --- | --- | --- | --- |
| **Pearson** | − 0.910** | − 0.928** | − 0.734** | 0.265 | − 0.925** | −0.127 | 0.308 |

** indicate correlation is sig0nificant at *p* < 0.01.

**Table S2** Alpha diversity index of bacterial and fungal

| Treatments | Bacterial | | | Fungal | | |
| --- | --- | --- | --- | --- | --- | --- |
|  | OTUs | Chao1 | Shannon | OTUs | Chao1 | Shannon |
| CK | 4008.67 ± 167.71 a | 4564.51 ± 243.46 a | 9.37 ± 0.27 a | 1560.00 ± 125.50 a | 6187.83 ± 447.91 a | 6.76 ± 0.55 a |
| NaHS200 | 4005.67 ± 114.92 a | 4381.37 ± 117.48 a | 9.31 ± 0.06 a | 1346.33 ± 139.23 a | 5197.75 ± 519.69 b | 6.09 ± 0.19 a |
| NaHS400 | 3068.33 ± 78.02 b | 3542.61 ± 56.88 b | 7.97 ± 0.27 b | 1050.00 ± 136.12 b | 4612.74 ± 509.97 bc | 5.30 ± 0.18 b |
| NaHS600 | 2261.00 ± 117.00 c | 2601.78 ± 164.18 c | 7.57 ± 0.48 b | 930.33 ± 14.29 b | 3655.60 ± 389.62 c | 4.45 ± 0.21 c |
| NaHS800 | 2476.33 ± 62.96 c | 2833.72 ± 94.92 c | 8.25 ± 0.09 b | 949.33 ± 22.85 b | 3844.66 ± 89.54 c | 4.82 ± 0.14 c |

All data are represented as the means ± SE. The different letters in the same column indicate significant differences at *p* < 0.05 according to LSD test.


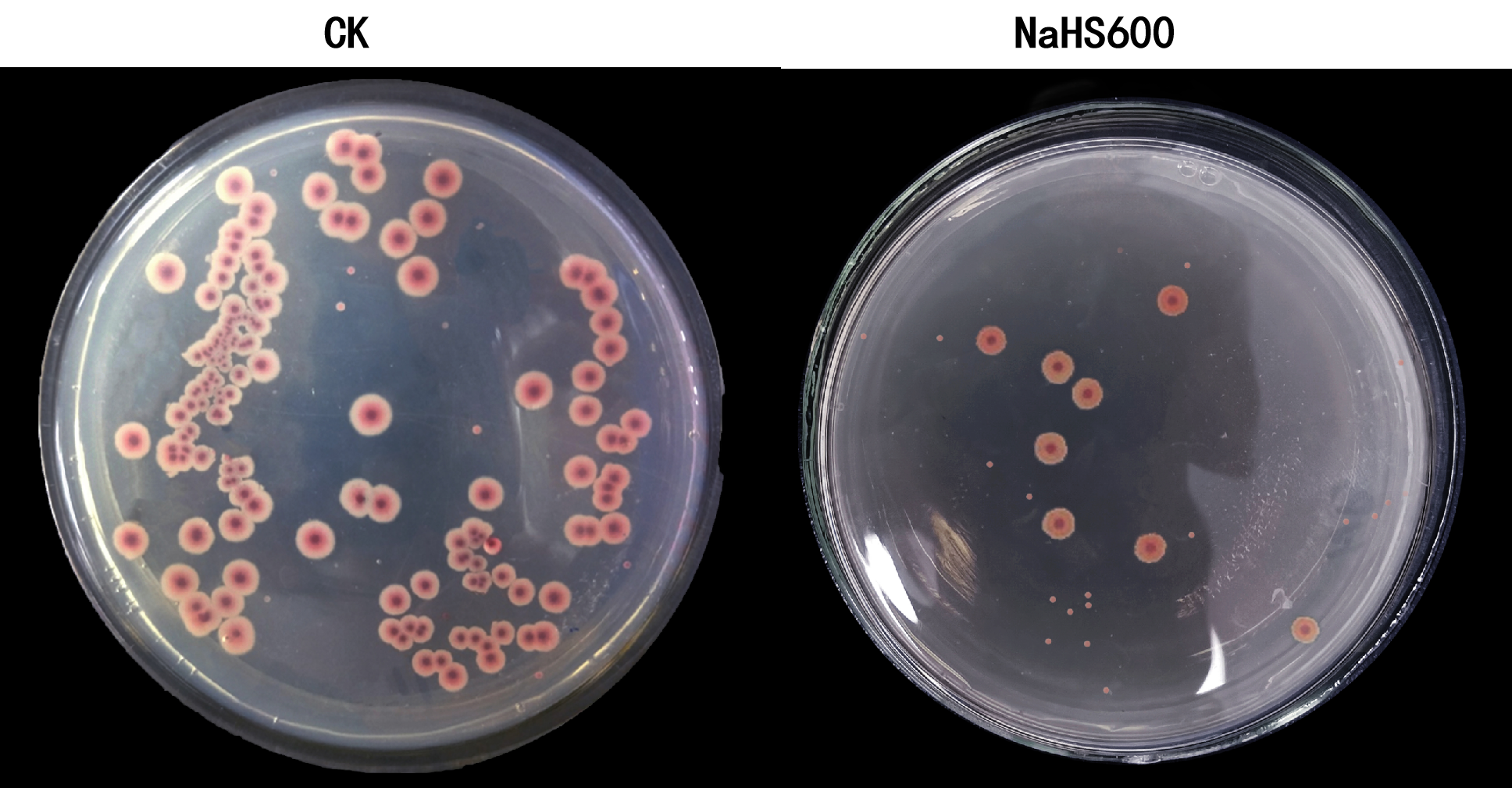


**Figure S1** The colony of *R. solanacearum* treated with 0 mg/mL (CK) and 0.6 mg/mL of NaHS (NaHS600).
